# Characterization and Biodegradation of Phenol by *Pseudomonas aeruginosa* and *Klebsiella variicola* Strains Isolated from Sewage Sludge and Their Effect on Soybean Seeds Germination

**DOI:** 10.3390/molecules28031203

**Published:** 2023-01-26

**Authors:** Samir A. Mahgoub, Shaza Y. A. Qattan, Salma S. Salem, Howaida M. Abdelbasit, Mohamed Raafat, Mada F. Ashkan, Diana A. Al-Quwaie, Ebtihal Abdullah Motwali, Fatimah S. Alqahtani, Hassan I. Abd El-Fattah

**Affiliations:** 1 Department of Agricultural Microbiology, Faculty of Agriculture, Zagazig University, Zagazig 44511, Egypt; 2 Department of Biological Sciences, Microbiology, Faculty of Science, King Abdulaziz University, Jeddah P.O. Box 80203, Saudi Arabia; 3 Department of Pharmacology and Toxicology, Faculty of Pharmacy, Umm Al-Qura University, Makkah 21955, Saudi Arabia; 4 Biological Sciences Department, College of Science & Arts, King Abdulaziz University, Rabigh 21911, Saudi Arabia; 5 Department of Biology, College of Science, University of Jeddah, Jeddah 21959, Saudi Arabia; 6 Department of Biology, Faculty of Sciences, University of Bisha, P.O. Box 551, Bisha 61922, Saudi Arabia

**Keywords:** phenol, biodegradation, *Pseudomonas*, *Klebsiella*, sewage sludge, soybean, germination

## Abstract

Phenols are very soluble in water; as a result, they can pollute a massive volume of fresh water, wastewater, groundwater, oceans, and soil, negatively affecting plant germination and animal and human health. For the detoxification and bioremediation of phenol in wastewater, phenol biodegradation using novel bacteria isolated from sewage sludge was investigated. Twenty samples from sewage sludge (SS) were collected, and bacteria in SS contents were cultured in the mineral salt agar (MSA) containing phenol (500 mg/L). Twenty colonies (S1 up to S20) were recovered from all the tested SS samples. The characteristics of three bacterial properties, 16S rDNA sequencing, similarities, GenBank accession number, and phylogenetic analysis showed that strains S3, S10, and S18 were *Pseudomonas aeruginosa, Klebsiella pneumoniae,* and *Klebsiella variicola*, respectively. *P. aeruginosa*, *K. pneumoniae,* and *K. variicola* were able to degrade 1000 mg/L phenol in the mineral salt medium. The bacterial strains from sewage sludge were efficient in removing 71.70 and 74.67% of phenol at 1000 mg/L within three days and could tolerate high phenol concentrations (2000 mg/L). The findings showed that *P. aeruginosa*, *K. pneumoniae,* and *K. variicola* could potentially treat phenolic water. All soybean and faba bean seeds were germinated after being treated with 250, 500, 750, and 1000 mg/L phenol in a mineral salt medium inoculated with these strains. The highest maximum phenol removal and detoxification rates were *P. aeruginosa* and *K. variicola.* These strains may help decompose and detoxify phenol from industrial wastewater with high phenol levels and bioremediating phenol-contaminated soils.

## 1. Introduction

Various living organisms, including microbes, plants, animals, and people, are highly vulnerable to phenol toxicity. Due to the expanding industrial sector, aromatic compounds are environmental contaminants in many places, such as freshwater, marine, and land [1]. Acute and high phenol concentrations can induce central nervous system abnormalities, cardiac depression, ocular irritation, edema, corneal bleaching, blindness, cardiovascular disorders, and gastrointestinal damage [2,3]. The primary pollutant in wastewater is the manufacture and utilization of phenol and phenolic compounds in industrial locations, such as oil refineries, coking plants, medicines, and plastics [4,5]. The maximum limit of phenol in the environment and tap water is 100 µg/L and 1–2 µg/L, respectively, based on WHO, because of its high toxicity [6].

Biodegradation is the ideal technique to dispose of phenol since it is inexpensive, environmentally beneficial, and simple to manage [7,8]. Nevertheless, various treatment approaches are available, including adsorption [9], solvent extraction [10], wet oxidation, and hydrogen peroxide. Fenton’s reagent, chemical oxidation, and combustion have removed phenol from contaminated samples [11,12]. However, these procedures are complicated, expensive, and not environmentally friendly [13,14]. Bacteria oxidize phenol into CO_2_ and H_2_O during metabolic processes [15] and use it as a sole source of carbon energy for development [16,17]; hence, these bacteria can degrade phenol [18].

Phenol is an artificial and naturally occurring aromatic compound and an essential intermediate in the biodegradation of natural and industrial aromatic compounds. Whereas many microorganisms capable of aerobic phenol degradation have been isolated, only a few phenol-degrading anaerobic organisms have been described to date. In this study, three novel nitrate-reducing microorganisms capable of using phenol as a sole source of carbon were isolated and characterized. Phenol-degrading denitrifying pure cultures were obtained by enrichment culture from sewage sludge. The three strains were shown to be different from each other based on physiologic and metabolic properties. Sequence analysis of 16S ribosomal DNA indicated that the phenol-degrading isolates were closely related to members of *Pseudomonas* and *Klebsiella.* In addition, the quantitative real-time PCR (RT-PCR) demonstrated the expression of all the genes required in the meta-cleavage pathway of phenol in three identified strains. The results of this study add three new members to the genus *Pseudomonas* and *Klebsiella*, which previously comprised only nitrogen-fixing species. So, discovering phylogenetically closely related species for phenol degradation is crucial; these strains can be exploited in real-world systems and aid in treating industrial pollutants [19,20,21]. The whole genome of *A. lwoffii* NL1 was sequenced, yielding 3499 genes on one circular chromosome and three plasmids. Enzyme activity analysis showed that *A. lwoffii* NL1 degraded phenol via the ortho-cleavage rather than the meta-cleavage pathway. The essential genes for phenol hydroxylase and catechol 1,2-dioxygenase were found to be separated by mobile genetic elements on a megaplasmid (pNL1); their function was verified by heterologous production in *Escherichia coli* and quantitative real-time PCR. [22]. Using these isolated microbes is more cost-effective and practical, reducing the environmental load by creating an efficient and cost-effective technology [23].

Microbial degradation has been considered an effective and eco-friendly technique for phenols removal. Various microorganisms isolated from contaminated sites can degrade phenols under aerobic conditions. The recent advances in the microbial treatment of phenol have improved our understanding of bioremediation [24,25]. *Acinetobacter calcoaceticus* can effectively treat phenolic wastewater. *A. calcoaceticus* effectively reduced 91.6% of phenol concentration (0.8 g/L) after two days and can tolerate phenol (1.7 g/L). These results revealed that *A. calcoaceticus* could effectively treat phenolic wastewater [26]. *A. lwoffii* NL1 could degrade 0.5 g/L phenol within 12 h and tolerate a maximum of 1.1 g/L phenol, and *A. lwoffii* NL1 can potentially be used for efficient phenol degradation in heavy metal wastewater treatment [22]. The bacteria of the genus *Pseudomonas* have been used as typical phenol-degrading microorganisms. *P. putida* can degrade phenol (1 g/L) in 162 h (6.17 mg/L per hour) [27], whereas *P. cepacia* isolated from industrial wastewaters can degrade 2.5 g/L phenol in 144 h (17.36 mg/L per hour) [28]. A new *Rhodococcus aetherivorans* strain has 35.7 mg/L per hour degradation rates at 0.5 g/L phenol [29]. The mutant M1 of *Rhodococcus ruber* SD3 can degrade 98% of 2 g/L phenol in 72 h by cell immobilization (27.2 mg/L per hour) [30]. The highest phenol degradation by *Serratia* sp. (accession number KT693287) was achieved at pH 7.5, the temperature of 30 °C and ammonium sulfate as a nitrogen source at the concentration of 0.4 g/L as well as a sodium chloride concentration of 0.15 g/L [31]. *Klebsiella oxytoca* degraded phenol (100 ppm) within 72 h. In comparison, at a phenol concentration above 400 ppm, the cells could not degrade the substrate efficiently due to the increasing phenol concentration in the medium. The optimum solution pH and temperature were 6.8 and 37 °C, respectively [32]. Aerobic phenol decomposition by bacterial strains requires many steps to increase the degradation rate and figure out how different physicochemical parameters and degradation processes affected the decomposition rate [33]. In addition, Sachan et al. [34] isolated two bacterial isolates from the effluent of a pulp and paper mill coded as SP-4 and SP-8 which showed luxuriant growth on phenol-amended minimal salt medium (MSM) in the presence of 1% glucose (*w*/*v*). In contrast, no growth has been observed in the absence of glucose. Both the strains showed fast and luxuriant growth at phenol concentrations of 0–1000 mg/L. These isolates can tolerate the phenol up to a phenol concentration of 1600 and 1800 mg/L, respectively. However, no growth has been observed in both bacterial isolates at a 2000 mg/L phenol concentration. Furthermore, *Pseudomonas putida* strains with intense degrading activity at considerable doses of phenol (8.5 mM) and chlorine-substituted derivatives such as O, M, P-chlorophenol (1.56 mM, 1.56 mM, and 2.34 mM, respectively) and 2,4-dichlorophenol (30 mg/L) have been described [27,35,36].

The temperature and pH are essential factors for bacterial growth and phenol degradation. In the typical mesophilic growth range of 20 °C to 30 °C, bacterial growth rates nearly double for every (10 °C) increase in temperature, and it does not alter between 35 and 40 °C. Still, the denaturation process for protein at higher temperatures decelerates growth rates for mesophilic microorganisms [37].

In vitro findings on phenol degradation have been carried out at an optimum temperature of 30 °C [38,39]; however, Tengku-Mazuki, [40] found that *Rhodococcus* sp. strain AQ5-14 has excellent potential for the bioremediation of phenol. The optimum conditions for phenol degradation (83.90%) were pH 7.0 and 0.4 g l-1 NaCl at 25 °C. In addition, S S, et al. [41] reported that no phenol degradation was noticed when the temperature was raised from 30 to 34 °C, indicating that phenol decomposition is a temperature-dependent process. Furthermore, Kotresha and Vidyasagar [42] found *P. aeruginosa* MTCC 4996 degraded phenol at temperatures ranging from 15 to 45 °C, with an optimal of 37 °C, and pH ranging from 6.0 to 10.0, with optimal of 7.0 to 7.5. Furthermore, Samimi and Moghadam [39] showed that the optimal condition for 97% phenol removal was pH 8, 35 °C, C: N ratio of 100: 30 (g/L), and salinity of 35 g/L.

Regarding pH factor, *Rhodococcus* sp. strain AQ5-14 preferred growth at the near-neutral condition. The optimum pH value for accurate phenol degradation was pH 7.0, which corresponded with their growth. The internal growth factors affecting an all-living cell are approximate. Most organisms cannot withstand pH below 4.0 or above 9.0 [40]. The acids or bases can easily penetrate bacterial cells since they prefer to live in a non-dissociated state under these conditions, and electrostatic forces cannot prevent them from entering cells [43].

All detected phenol-degrading isolates in the previous studies can tolerate phenol concentrations less than 2g/L. Therefore, in this study, we isolated, characterized, and evaluated a novel phenol-degrading bacteria (*Pseudomonas aeruginosa, Klebsiella pneumoniae,* and *Klebsiella variicola*) from sewage sludge (SS). The capability of the bacterial strains to degrade and detoxify phenols at a high level > (2 g/L) and their effect on bean seed germination have been evaluated.

## 2. Results and Discussion

### 2.1. Isolation of Phenol-Degrading Bacteria

**Figure 1:**
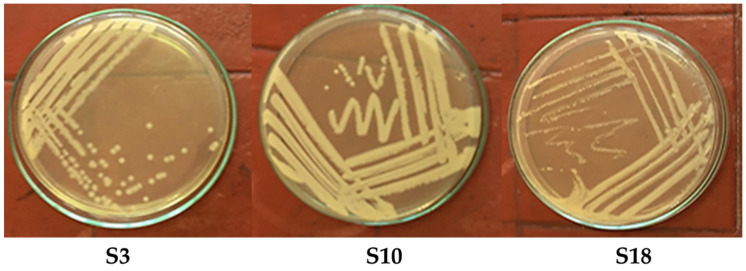
Bacterial growth of *Pseudomonas aeruginosa* (S3), *Klebsiella pneumoniae* (S10), and *Klebsiella variicola* (S18) onto MSM plates amended with 500 mg/L phenol.

Table 1 shows the OD of bacterial growth in MSM supplemented with different phenol concentrations (0.0, 500, 750, 1000, 1500, 2000 mg/L) at 600 nm. The sewage sludge sample (10 g) was added aseptically to 90 mL of MSM media supplemented with different levels of phenol and incubated in a shaking incubator (120 rpm; 35 °C) [38]. The culture broths were examined at an OD_600_ nm after incubation for three days. The noticeable increase in optical density (OD) in the control compared to all the samples. The higher value of OD was obtained from the SS3, SS10, and SS18 compared to the control at all the phenol concentrations. Thus, from these samples, 10 mL of the culture broth was transferred to a new shaking flask containing 90 mL of fresh MSM containing 500 mg/L phenol and incubated for three days. This culture broth was streaked onto MSM agar plates containing 0.5 g/L phenol (Figure 1). After 3–4 days of incubation at 35 °C, colonies on agar plates were harvested and subcultured repeatedly to obtain pure cultures [39]. The bacterial isolates of S3, S10, and S18 were stored in LB agar slants at (4 °C) for the following experiments.

### 2.2. Identification and Characterization of Phenol-Degrading Bacterial Isolates

The samples (10 mL) were put into a mineral salt broth containing 500 mg/L phenol to enrich phenol-degrading bacteria in a 250-mL flask. The phenol-utilizing bacterial isolates were subsequently grown on mineral salt agar (MSA) containing 500 mg/mL of phenol. All colonies that develop on the phenol-containing solid medium can use phenol as their primary carbon and energy source. These isolates displayed more significant growth in phenol-containing media containing up to 2 g/L phenol (Table 2). Regarding the physical and biochemical properties, Gram staining, motility, acid formation of glucose, oxidase, catalase, oxidative fermentation, and growth under different conditions were on these bacteria. The bacterial strains S3, S10, and S18 from sewage sludge samples may tolerate phenol concentrations up to 2000 mg/L. Among the twenty established isolates, these strains were shown to be the most effective in terms of phenol tolerance and incubation period (Table 2). Using these isolated microbes is more cost-effective and practical, reducing environmental load by developing efficient and cost-effective technologies [21]. These bacteria are essential to soil and capable of degrading polycyclic aromatic hydrocarbons. Still, it is frequently identified in water reservoirs contaminated by animals and people, such as sewage and sinks in and around hospitals.

Table 3 revealed that all bacteria were G^−^ rods, aerobic, motile, positive for oxidase- and catalase, and produced acid from glucose. Three isolates were Gram-negative, strictly aerobic, motile, and rod-shaped. Table 3 shows the colonies’ growth and morphological, microscopic, and biochemical features. The detected bacteria, *Pseudomonas* spp., and *Klebsiella* spp., were aerobic, positive for oxidase and catalase, and produced acid from glucose (Table 3). The phenol-degrading isolates S3, S10, and S18 could grow at temperatures ranging from 25 to 40 °C and pH levels of 5 to 9. The optimal growing conditions were between 30 and 35 °C and pH 7.0. In this work, three Gram-negative isolates were plated on a solid MSM medium supplemented with different levels of phenol (Figure 1). The species of *Pseudomonas* and *Klebsiella* have been characterized by Stover et al. [44]. *Pseudomonas* and *Klebsiella* species can typically thrive on MSM medium containing a single carbon and energy source. *P. aeruginosa* develops well around 37 °C. However, it can also survive in a wide range of temperatures between 4 and 42 °C. *Pseudomonas* and *Klebsiella* species may decompose phenol [35,45].

### 2.3. 16S rDNA Gene Sequence Analysis of the Three Isolates

**Figure 2:**
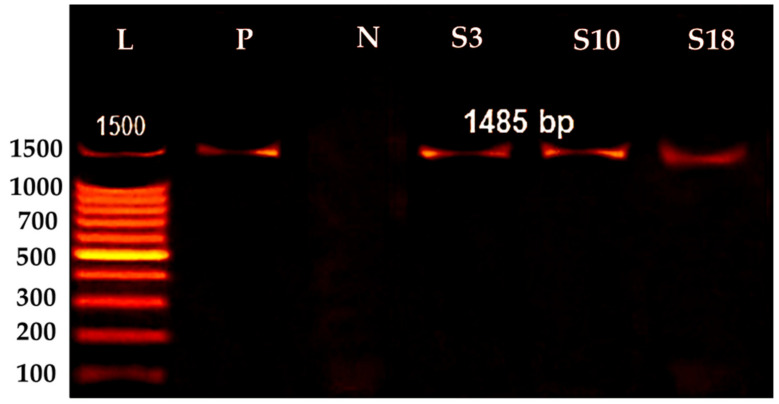
16S rRNA genes of bacterial isolates, Lane 1, Ladder [(L) 100–1500 bp], Positive control (P, *Pesudomonas areginosa*), Negative control (N, *Aeromonas hydrophila*) identified isolates (S3, S10, and S18) at (1500 bp).

Figure 2 shows that each isolate was identified based on its morphology and 16S rDNA gene sequence analysis. The development of a single band in the PCR result of the isolates (Figure 2) suggests that PCR effectively amplified the 16S rRNA gene sequence at 1500 bp.

In this work, 16S ribosomal DNA sequence analysis revealed that phenol-degrading isolates were closely related to *Pseudomonas* and *Klebsiella* species. In addition, quantitative real-time PCR (RT-PCR) revealed the expression of all genes necessary for the phenol meta-cleavage process in three discovered strains. The results of this study add three new members to the genera *Pseudomonas* and *Klebsiella*, which previously constituted exclusively nitrogen-fixing bacteria. Therefore, it is essential to discover phylogenetically related species for phenol degradation; these strains may be utilized in real-world systems and help treat industrial contaminants [19,20,21]. *A. lwoffii* NL1’s whole genome was sequenced, revealing 3499 genes on one circular chromosome and three plasmids. *A. lwoffii* NL1 degraded phenol by the ortho-cleavage pathway rather than the meta-cleavage pathway, as determined by enzyme activity analysis. Critical genes for phenol-hydroxylase and catechol-1,2-dioxygenase were identified on a giant plasmid (pNL1) and were discovered to be separated by mobile genetic elements; their activity was verified by heterologous production in *Escherichia coli* and quantitative real-time PCR [22].

The 16S rRNA gene sequences for *Pseudomonas aeruginosa* ON880415.1, *Klebsiella pneumoniae* ON0414.1, and *Klebsiella variicola* ON880418.1 have been submitted to GenBank. All sequencing results were compared to the 16S rRNA gene sequence in the NCBI GenBank database. Ten (50%) of the identified bacteria were *Pseudomonas* spp., and ten (50%) were *Klebsiella* spp. Based on consensus sequences for the 16S rRNA gene, a phylogenetic tree was created by comparing sequences from 29 GenBank strains to strain S3 and typical Gram-negative *Pseudomonas* spp.

The blast was used to compare our data to reference sequences from the NCBI Gene Bank Data Base. The phylogenetic analysis revealed that the 29 phenol-degrading bacteria had identical 16S rDNA gene sequences and belonged to the same cluster. This cluster’s members showed a significantly greater specificity and could be separated into two distinct subclusters. The *Pseudomonas* species exhibited tighter relationships, with a bootstrap score between 98 and 99%, confirming their placement within *P. aeruginosa* ON88415.1 (S3). In addition, sequences from 16 GenBank strains were compared to those of strains S10 and S18 and typical Gram-negative *Klebsiella* species. *Klebsiella* spp. has a bootstrap value between 97 and 89% and was well-established within two clusters. Cluster 1 is comprised of *K. pneumonia*, while Cluster 2 is comprised of *K. variicola*. Global alignment was utilized for pairwise sequence alignment and the generation of similarity values in the EzBio-Cloud database [46].

**Figure 3:**
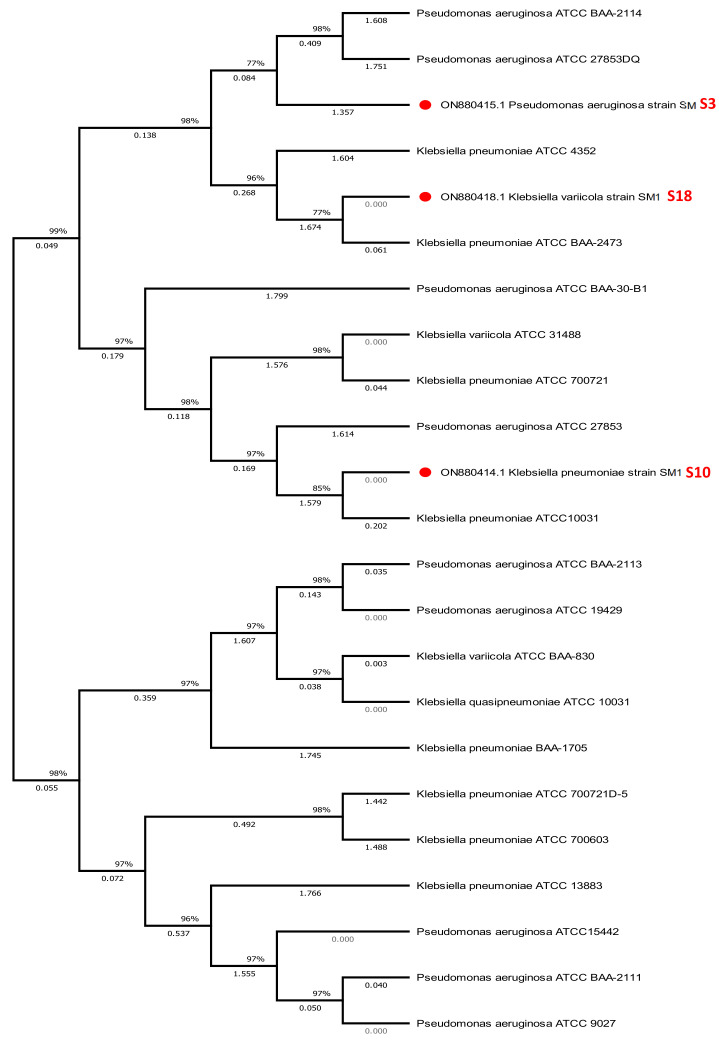
Phylogenetic tree of *Pseudomonas aeruginosa* (S3), *Klebsiella pneumoniae* (S10) and *Klebsiella variicola* (S18).

This molecular taxonomic investigation determined the strains to be *Pseudomonas aeruginosa* (S3), *Klebsiella pneumoniae* (S10), and *Klebsiella variicola* (S18). The evolutionary history was inferred using the neighbor-joining and maximum likelihood techniques with 1000 repetitions of MEGA version 7’s bootstrap values [40]. The nucleotide sequence from this study has been added to the NCBI nucleotide sequence databases with the Accession numbers: ON880415.1, ON880414.1, and ON880418.1, respectively, and these strains are 97–99% similar to the strains in the GenBank, *P. aeruginosa* JN995663.1, *K. pneumoniae* MT516162.1, and *K. variicola* ON597432.1, respectively (Figure 3).

### 2.4. Evaluation of the Bacterial Isolates for Phenol Degradation

**Figure 4:**
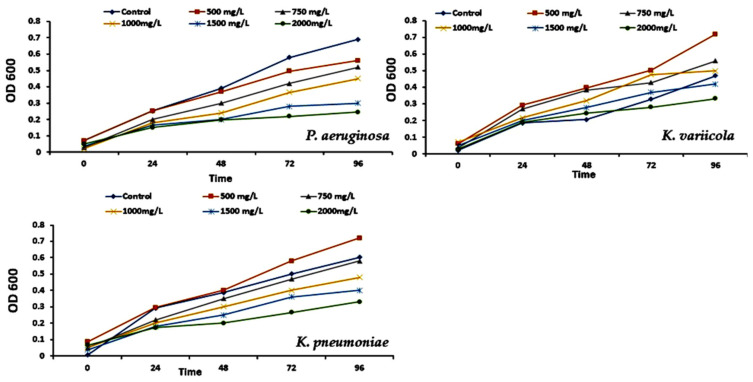
Effect of different phenol concentrations (0, 500, 750, 1000, 1500, and 2000 mg/L) on the growth of *Pseudomonas aeruginosa*, *Klebsiella pneumoniae,* and *Klebsiella variicola*.

This research aims to isolate particular bacteria from a diverse population of naturally existing microorganisms. Twenty isolates were selected on LB media after 24 h incubation. After 24 h of growth on LB agar plates containing 100 µL of a 10^3^–10^6^-fold dilution of the EM after three weeks of enrichment and one week of bacterial isolation. These isolates were evaluated for growth in MSM supplemented with varying amounts of phenol (Table 1). The phenol-degrading abilities of *P. aeruginosa* (S3), *K. pneumoniae* (S10), and *K. variicola* (S18) at different starting phenol concentrations (0.0, 500, 750, 1000, 1500, and 2000 mg/L) were studied by measuring the phenol concentration and cell growth at OD_600_ (Figure 4).

**Figure 5:**
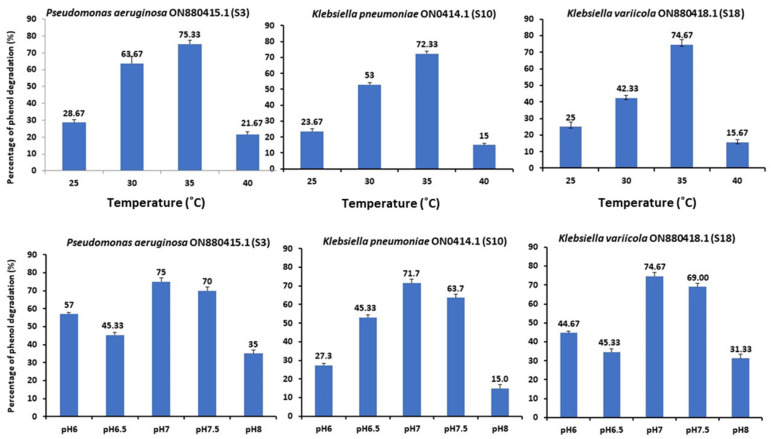
Effect of pH and temperature on phenol biodegradation by *Pseudomonas aeruginosa* (S3), *Klebsiella pneumoniae* (S10), and *Klebsiella variicola* (S18) (initial phenol concentration was 1000 mg/L).

Maximum phenol degradation was reported at an initial 1000 mg/L value (Figure 5). The three strains survived at phenol concentrations up to 1500 mg/L with a degradation rate of 39.82%. Concerning the pH and temperature, the bacterial strain can populate at a pH range of 6–8 to degrade 75% of phenol (Figure 5). Figure 5 demonstrates the bacterial growth of strains S3, S10, and S18 and the 75% phenol breakdown at 35 °C. Conversely, the phenol degradation decreased substantially at temperatures between 25 and 40 °C. Therefore, the ideal growing temperature for strains S3, S10, and S18 was 35 °C. Shourian et al. [47] and Kotresha and Vidyasagar [42] discovered that *Pseudomonas aeruginosa* could degrade phenol at temperatures ranging from 15 °C to 45 °C, with an optimal of 37 °C and pH values ranging from 6.0 to 8.0, with an optimal of 7.0 to 7.5 (phosphate buffer). The optimal temperature and pH levels were 35 °C and 7.0, respectively. The mechanism of phenol degradation by isolated strains can be correlated with these pathways. The P-nitro phenol degradation pathway involves the formation of benentroid, which contains aromatic ring cleavage that results in the biodegradation of P-nitro phenol. The p-nitro phenol was degraded to benzoquinone (BQ) and hydroquinone (HQ) by the Arthobacter strain [48]. Gram-negative bacteria, such as Burkholderia spp. and Moraxella spp., convert hydroquinone into maleyl acetate [49]. While G+ bacteria, such as *Bacillus* spp. and *Arthrobacter* spp., converted 4-nitro-phenol into 4-nitro catechol and hydroxyquinol [50]. Consequently, several studies conducted on 4-nitro phenol degradation have been reported. In association with the hydroquinone pathway, the 4-nitro phenol degradation genes were cloned from the *Pseudomonas* sp. strain and *Pseudomonas putida*. In the case of the hydroxyquinol pathway, no 4-nitro phenol catabolic gene has been reported. The individual nucleotide sequence of 4-nitro phenol degradation genes (nphA1A2) was reported using *Rhodococcus* sp. strain PN1 [51].

Peptone and yeast extract are the most effective organic nitrogen sources for phenolic growth and breakdown. In addition, a maximum of 98.93% phenol degradation was observed at an initial concentration of 1000 mg/L at 35 °C and pH 7.0 for 96 h [52], in agreement with previous studies that stated *P. aeruginosa* MTCC 4997 strain isolated from effluents of petrochemical companies completely degraded phenol at 15–45 °C range, with the optimal temperature being 37 °C [42]. Mishra and Kumar [53] found that *P. aeruginosa* KBM13 needs 40 °C to degrade the phenol in the medium. The susceptibility of these bacteria to higher phenol concentrations may require phenol acclimation before its destruction [54].

Concerning time and acclimatization, it has also been discovered that bacterial growth develops in the phenol medium, showing that the bacteria eventually adapt to the compound. Sachan et al. [34] isolated competent bacterial cells from phenol-formaldehyde resin factory effluent. Numerous studies suggest that the pH range (7–10) was optimal for the growth and phenol degradation of *Pseudomonas* sp., based on the bacterial origin [55,56]. Mishra and Kumar [53] demonstrated that the optimal pH for *P. aeruginosa* to degrade phenol was eight. For instance, the optimal pH range for *Klebsiella oxytoca* to degrade phenol was 6.8 [57]. The pH’s influence on phenol decomposition may be attributable to its effects on transport, stimulation of enzyme activity, and nutrient solubility [58]. The pH substantially affects the metabolic processes necessary for phenol breakdown. According to reports, the pH influences the surface charge of the activated sludge biomass cells [59].

**Figure 6:**
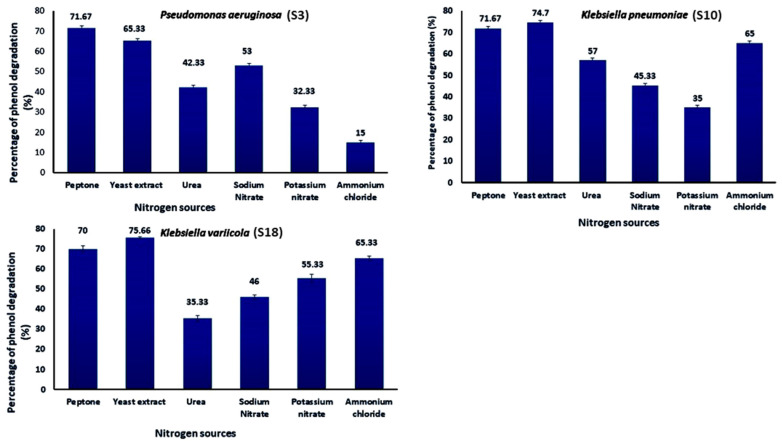
Effect of N sources on phenol biodegradation by *Pseudomonas aeruginosa* (S3), *Klebsiella pneumoniae* (S10), and *Klebsiella variicola* (S18) (initial phenol concentration was 1000 mg/L).

As shown in Figure 6, the effect of various nitrogen sources on deterioration has been examined. The influence of three organic nitrogen sources, peptone, yeast extract, and urea, and three inorganic nitrogen sources, sodium nitrate, potassium nitrate, and ammonium chloride, on the development and breakdown of phenol by Strains S3, S10, and S18 were considered. The N sources were added to the MS medium containing (1 g/L) phenol and incubated for four days at 35 °C and pH 7 in triplicate at a temperature of 35 °C. The choice of nitrogen supply influenced the development of S3, S10, and S18 strains and phenol degradation. More than 75% of the nitrogen sources, peptone and yeast extract, demonstrated strong growth and phenol degradation rates. The addition of yeast extract increased the percentage of phenol degradation to 75%, whereas urea resulted in the lowest growth and phenol degradation (35%). Consequently, yeast extract was the best of the evaluated organic nitrogen sources for maximal growth and phenol degradation.

The present investigation proved that adding yeast extract to the medium resulted in the maximum phenol elimination efficiency and promoted the development of strain S3. The nature of yeast extract, easily obtainable as amino acids in a medium containing mineral salts, is needed for the phenol breakdown process by bacterial cells [60,61]. Mishra and Kumar [53] have shown that yeast extract is the optimum nitrogen source for growth and phenol decomposition. Ammonium sulfate was established to be the best nitrogen source at a concentration of 0.4 g/L and a sodium chloride concentration of 0.15 g/L. The different nitrogen sources, such as peptone and yeast extract, were used to get the optimum growth conditions for phenol degradation [62,63], which were selected to do a single factor test for further study of the optimum conditions for bacterial growth, which was an ability to degrade phenol. *K. pneumoniae* (S10) and *K. variicola* (S18) showed maximum phenol degradation in the presence of yeast extract at pH 7. However, in the case of *P. aeruginosa* (S3), peptone was the best nitrogen source of phenol degradation. Replacement of yeast extract with tryptone and urea affected phenol degradation efficiency [64]. The phenol biodegradation rate may significantly affect by the presence of a high load of nitrogen pollutants. Ammonia is known to be toxic to aquatic life and creates an excellent oxygen demand in receiving waters. The highest phenol degradation by *Serratia* sp. (accession number KT693287) was achieved at pH 7.5, the temperature of 30 °C and ammonium sulfate as a nitrogen source at the concentration of 0.4 g/L as well as a sodium chloride concentration of 0.15 g/L [31].

The microbial phenol degradation, with an emphasis on aerobic degradation utilizing bacterial strains, various strategies for improving/enhancing the phenol degradation rate, impacts of various physicochemical parameters on the degradation process, and degradation processes [33]. The three bacterial strains, S3, S10, and S18, were cultured on MSM medium with phenol (1000 mg/L) at a temperature of 35 °C and a pH of 7 (phosphate buffer). This combination was kept in Erlenmeyer flasks of 250 mL capacity. The cultures were shaken on a shaker (120 rpm) at the specified temperature. The phenol degradation was assessed at 96 h using HPLC and is presented in Supplementary Figure S1).

### 2.5. Effect of K. variicola on Soybean Seedlings

We conducted a trial on three isolates as phenol degraders, we found that S3 and S18 were more effective in phenol degradation, so we tested these isolates (S3 and S18) with a seed germination test in phenol.

**Figure 7:**
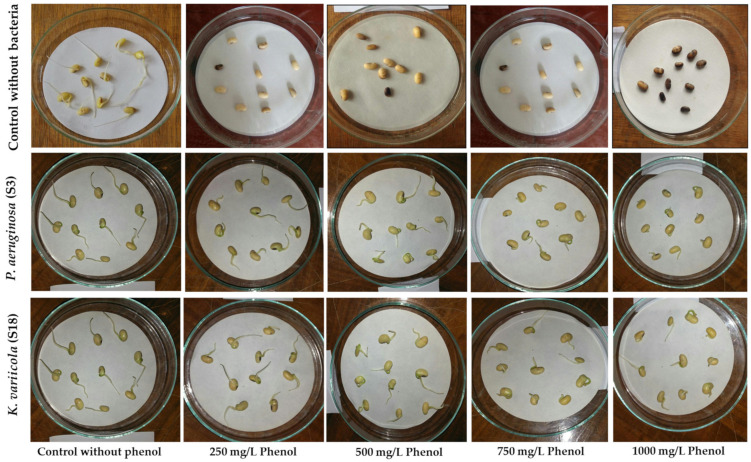
Effect of *Pseudomonas aeruginosa* (S3) and *Klebsiella variicola* (S18) on soybean seed germination after bioremediation and detoxification of water contaminated with different concentrations of phenol (0.0, 250, 500, 750, and 1000 mg/L).

Seed germination results (Figure 7 and Table 4) showed higher inhibitory activity of the different phenol concentrations. According to germination percentage, the group observed: control without bacteria and treated with different phenol concentrations (250 to 1000 mg/L) showed total inhibition in seed germination. In contrast, group *Pseudomonas aeruginosa* and group *Klebsiella pneumonia* in the presence of phenol concentrations (up to 1000 mg/L) showed 100% germination. The seeds in the control without bacteria and treated with all phenol concentrations appear dark in color. The dark seeds increase as phenol concentration increases, as shown in Table 4 and Figure 7. Thus, soaked soybean seeds in 250 up to 1000 mg/L phenol stress significantly decreased (*p* < 0.05) the percentage of germination and the seedling growth rate in the soybean seedlings and inhibited the germination by 100%. However, using the bacterial suspension of *P. aeruginosa* and *K. variicola* significantly increased (*p* < 0.05) the percentage of germination and the seedling growth rate in the soybean seedlings in the presence of phenol stress.

In a saline-alkaline environment, *K. variicola* promoted the development of maize seedlings effectively [65]. In our experiment, primary roots reached a maximum length (2.83 ± 0.65 cm) in the control and 2.89 ± 0.32 in the *K. variicola*. The primary roots in the *P. aeruginosa* group range from 2.83 ± 0.45 cm at 250 mg/L to 2.41 ± 0.24 cm at 1000 mg/L. As discovered in spinach, phenol can modify mitochondrial and chloroplast membranes, impeding the energy transfer required for ion transport. Singh et al. [62] demonstrated that 100 µM of caffeic acid applied to the soil completely inhibited the germination of mung bean seedlings. Plant height and the fresh weight of shoots dropped as phenolic chemical concentrations in the soil increased. At doses of 50 and 100 mg/L, phenol, coumarin, ferulic acid, and naringenin inhibited soybean seed germination and the development of seed-borne fungus significantly. *P. aeruginosa* (S3) and *K. variicola* (S18) have the capacity to remove phenol. Based on a separate investigation, *K. variicola* may generate indole acetic acid. According to the studies, indole acetic acid is one of the primary hormones regulating the growth and development of plant cells, including cell proliferation and division, organ development, and directional movement [66,67].

*K. variicola* played a role in indole acetic acid, acetoin, ammonia, phosphorus, potassium production, and nitrogen fixation. A high level of colonization was observed in the rhizosphere soil of maize seedlings. Following the application of *K. variicola* in neutral and saline-alkali soils, the nutrient composition of the rhizosphere soil of maize seedlings increased in varying degrees, more notably in saline-alkali soil. The content of organic matter, alkali-hydrolyzable nitrogen, available phosphorus, available potassium, alkaline phosphatase, sucrase, urease, and catalase increased by 64.22%, 117.39%, 175.64%, 28.63%, 146.08%, 76.77%, 86.60%, and 45.29%, respectively, in saline-alkali soil [65]. Thus, *K. variicola* could be a promised strain with more biological functions, such as removing phenol from our study.

## 3. Material and Methods

### 3.1. Methods

The minimal salt medium (MSM) and Luria–Bertani (LB) medium were used in this study. MSM consisted of 10 g of NaCl, 0.42 g of MgSO_4_.7H_2_O, 0.29 g KCl, 0.83 g KH_2_PO_4_, 1.25 g Na_2_HPO_4_, 0.42 g NaNO_3_, and 20g agar in one liter of distilled water, while the LB medium consisted of NaCl (5g), tryptone (10 g), and yeast extract (5 g) g/L [34]. The media was autoclaved for 15 min at 121 °C and 15 pounds per square inch (psi) and then cooled. The MSM medium was supplemented with phenol or other nitrogen sources. The pH was adjusted (pH 6.8–7.0), and various phenol concentrations (0.5–2 g/L) were added. A 0.45 µm filter syringe was used to add 50 µg/L phenol directly from a stock of phenol to medium working solutions. On a rotary shaker (120 rpm), cells were incubated in flasks at 35 °C at 120 rpm. Periodically, samples were collected to determine bacterial growth and phenol concentration.

### 3.2. Sample Collection and Sources of Bacterial Isolates

The phenol-degrading bacterial isolates were obtained from sewage sludge (SS) at SWTP, Egypt. Before usage, the sludge was kept in sealed containers at 4 °C. The sample collection occurred between May and October of 2021. First, 10 g of SS were weighed into 90 mL of sterile peptone saline (8.5 g/L NaCl) to generate 10^−1^ dilution. The mixture was agitated for one minute to facilitate the separation of microbial cells from sludge particles. One mL (1 mL) from the 10^−1^ dilution was transferred to a sterile test tube containing 9 mL of sterile physiological saline to prepare a 10^−2^ dilution. One mL was transferred into a 9 mL sterile physiological saline to achieve the 10^−3^ (always use a pipette to transfer). The content of the 10^−3^ dilution test tube was shaken, and dilution continued until the 10^−8^ dilution was obtained. After that, an aliquot of 0.1 mL of 10^−5^, 10^−6^, 10^−7,^ and 10^−8^ dilutions were plated in triplicates on mineral salt agar (MSA) supplemented with 500 mg/mL of phenol. The plates were then incubated at 30 °C for 24 h for bacterial growth. The pure colonies have been taken and purified onto LB agar plates.

### 3.3. Isolation of Phenol-Degrading Bacteria

Each bacterial culture (10 mL) from SS1 up to SS20 samples was added aseptically to 90 mL of MSM media supplemented with different levels of phenol (0.5–2 g/L) and incubated in a shaking incubator (120 rpm; 35 °C) [68]. The optical density of the bacterial cultures was examined at a wavelength of 600 nm throughout the incubation period. After 2–3 days of a noticeable increase in optical density (OD), 10 mL of the culture broth was transferred to a new shaking flask containing 90 mL of fresh medium. The final culture broth was serially diluted and distributed on LB agar and MSM agar plates containing 0.5 g/L phenol. After 3–4 days of incubation at 35 °C, colonies on agar plates were harvested and subcultured repeatedly to obtain pure cultures. A microscopic examination confirmed the cleanliness of the artifact. By optical microscopy, the morphological features of the isolated colonies were examined. Gram staining, motility, starch hydrolysis, and gelatinase activity are common physio-chemical characteristics of bacterial strains that were carefully determined using Bergey’s manual [69]. As indicated earlier, the indole, methyl red, and hydrogen sulfide tests were also conducted [70]. All bacterial isolates were selected for phenol removal assays at various phenol concentrations (0.0, 0.5, 1, 1.5, and 2 g/L) by Klibanov et al. [71]. S3, S10, and S18 isolates were stored in LB agar slants at 4 °C and in LB supplemented with 20% sterile glycerol at 20 °C.

### 3.4. Characterization of the Most Effective Bacterial Isolates by 16S rDNA and Phylogenetic Analysis

The genomic DNA of bacterial cells was recovered by phenol-chloroform after treating them with achromopeptidase, lysostaphin, and mutanolysin [72]. DNA was separated by electrophoresis in a 0.7 or 1.5% agarose gel with TBE buffer, stained with ethidium bromide, and seen under ultraviolet light. DNA fragment sizes were determined by comparing them to a 50 bp ladder (Fermentas). PFf 5′ AGGGATGTATTTATTAGATAAAAAATCAA 3′ and PFr 5′ CGCAGTAGTTTCTTCAGTAAATC 3′ are the primer sequences used for PCR amplification. 16S rRNA gene was amplified by PCR and then partially sequenced by Mannerová et al. [73]. The gene sequence was rebuilt using RNAmmer version 1.2 from whole-genome shotgun (WGS) data [74] and compared to other *Pseudomonas* and *Klebsiella* species. In the EzBioCloud database, a global alignment technique allows pairwise sequence alignment and the creation of similarity values [46]. The evolutionary history was estimated using the neighbor-joining and maximum likelihood techniques with 1000 bootstrap values from MEGA version 7 [75].

### 3.5. Preparing for Bacterial Inoculation

Each strain of *P. aeruginosa* (S3), *K. pneumoniae* (S10), and *K. variicola* (S18) was inoculated into LB broth, and the culture flasks were incubated at 35 °C and 120 rpm for 24 h in a shaker incubator. The bacterial culture was centrifuged for 15 min at 6000× *g*; the residue was obtained, washed, and resuspended in sterilized distillate water. This procedure was carried out three times. The sample was diluted to 1 × 10^2^, 1 × 10^4^, and 1 × 10^6^ CFU/mL, following the experiment’s specifications.

### 3.6. Evaluation of the Three Bacterial Isolates on Phenol Degradation

Referring to the one variable at a time (OVAT) technique, all factors were held constant in these experiments, with only the targeted variable being changed to optimize process variables. Briefly, the optical density of prepared cultures of each isolate, S3, S10, and S18, were adjusted at (1.0) at a wavelength of 600 nm. The inocula (2%) were inoculated into the phenol (1 g/L)-MSM medium as the only carbon source. The bacterial growth was monitored at 0, 1, 2, 3, and 4 days. Additionally, phenol degradation was recorded after four days of incubation in a shaking incubator (120 rpm) at different conditions of temperatures (25, 30, 35, and 40 °C), pH values (6, 7, and 8), and different nitrogen sources in growth medium MSM that contain peptone, yeast extract, urea extract, NaNO_3_, KNO_3_, and NH_4_Cl at a concentration of 2 g/L. Buffer solutions with different pH ranges, i.e., acetate buffer for pH 6.0, phosphate buffer for pH 6.5, 7.0, and 7.5, and Tris-HCl buffer for pH 9 were used to achieve different pH values [31].

The phenol concentrations were measured at 520 nm using 4-amino antipyrine following American Public Health Association-recommended procedures [6]. In summary, 0.3 mL of 2% aqueous 4-amino antipyrine solution and 1 mL of 2 N NH_4_OH were added to a 5 mL sample. After adequately mixing the contents, 1 mL of 2% K_3_FeCN_6_ is added. At pH 10, the OH group reacts with 4-amino antipyrine and K_4_Fe(CN)_6_ to produce a purple-red color. Quantitative analysis of phenol degradation was conducted by measuring the phenol content in the supernatant of each sample and calculating degradation using the following equation

% Degradation = (A − B)/A × 100

The phenol content was determined by comparing the absorbance at 520 nm to the phenol standard curve. Where A is the initial concentration of phenol (Zero h. sample), and B is the phenol concentration in the sample taken at various time intervals.

### 3.7. Phenol Degradation Assay by HPLC Analysis

HPLC (Agilent 12,000 series) detected the phenol degradation in MSM by three new bacterial strains. The separation column was C18 column (Eclipse Plus, 4.6 mm × 100 mm i.d., 3.5 m), and the mobile phase was composed of 0.01% formic acid in water (A) and 0.01% formic acid in acetonitrile (B) with a flow rate of 1 mL/min. The mobile phase was sequentially programmed as follows: 0 min (85% A); 0–5 min (50% A); 5–6 min (10% A); 6–7 min (10% A); 7–7.5 min (85% A); and 7.5–10 min (85% A). The standard solutions and samples were injected at a volume of 40 µL, the column temperature was kept at room temperature, and the phenol degradation rate was monitored at 280 nm [76]. The elutes were identified by comparing their retention time to the reference compound.

### 3.8. Effect of Pseudomonas Aeruginosa (S3) and Klebsiella Variicola (S18) on Soybean Seed Germination

Bean, soy, and faba bean seeds (270 days old) of the same size and without damage were picked, cleaned, soaked, and sterilized with a 10% sodium hypochlorite solution for 10 min; then, the seeds were immersed for six hours in distilled water, then washed several times and dried. The seeds were then placed in an incubator set at 25 °C. A piece of filter paper was used as a negative control, and sterile water was gently poured over it until it was slightly moist. The seeds covered with bacterial suspension were used as the positive control group. The bottom of the germination plate (20 cm) was lined with filter paper. Ten soaking seeds were distributed uniformly on each plate (20 cm). A piece of filter paper was put on top of the seeds and evenly coated with 3 mL of each bacterial suspension grown in MSM containing 1g/L phenol (1.4 × 10^8^ CFU/mL). The germination box was covered with a lid and incubated at 25 °C for 24 h to speed up germination in the dark.

### 3.9. Statistical Evaluation

Experiments to determine the biodegradation capability of the studied strain were conducted in triplicate, and the findings presented in the figures and tables were the means. The figures shown display the mean and standard deviation values for three replicates. The means of triplicate data were analyzed by ANOVA test, and the LSD test was used as a post-doc test to compare the means.

## 4. Conclusions

The phenol-degradable bacterial strains of interest were isolated and identified from sewage sludge. The 16S rDNA sequencing and the phylogenetic analysis revealed that the isolates were found to be *Pseudomonas aeruginosa* (S3) ON88415.1, *Klebsiella pneumoniae* (S10) ON880414.1, and *Klebsiella variicola* (S18) ON880418.1. *P. aeruginosa* (S3), *K. pneumoniae* (S10), and *K. variicola* (S18) can thrive in a liquid medium with varying amounts of phenol as the only carbon and energy source (0, 250, 500, 750, 1000, 1250, 1500, and 2000 mg/L). The strains could digest 75.66% of phenol (1000 mg/L while these strains were resistant and tolerant in the presence of phenol up to 2000 mg/L. The ideal growing conditions for each bacterium to degrade phenol were 35 °C, pH 7.5, and the best nitrogen source was peptone and yeast extract. Because native microbial species in polluted settings were more adaptable than non-indigenous microorganisms, their dominance aided the bioremediation of phenol-contaminated ecosystems. *K.variicola* consummated phenol through removal from media to promote soybean seedlings under phenol stress. *K. variicola* (S18) strains are promising and might be employed for the bioremediation of phenol-contaminated regions. *K. variicola* could be a novel strain with more biological function which is friendly to the ecosystem. *K. variicola* could be applied for the integrated biological treatment of wastewater for the removal of phenol from water.

## Figures and Tables

**Table 1:** Bacterial populations (OD600 ± SD) from sewage sludge (SS) can grow on MSM supplemented with different concentrations of phenol (500 to 2000 mg/L).

Samples	Concentrations of Phenol (mg/L)					
	Control	500	750	1000	1500	2000
*SS1	1.34 ± 0.41	0.75 ± 0.23	0.43 ± 0.37	0.22 ± 0.28	−	−
SS2	1.38 ± 0.43	0.96 ± 0.26	0.77 ± 0.36	0.42 ± 0.26	−	−
SS3	1.45 ± 0.42	1.38 ± 0.34	1.29 ± 0.35	1.12 ± 0.22	0.89 ± 0.23	0.37 ± 0.32
SS4	1.22 ± 0.39	0.87 ± 0.33	0.67 ± 0.34	0.31 ± 0.21	−	−
SS5	0.98 ± 0.43	0.76 ± 0.35	0.43 ± 0.32	−	−	−
SS6	0.87 ± 0.37	0.49 ± 0.34	0.47 ± 0.37	0.33 ± 0.28	−	−
SS7	0.93 ± 0.36	0.47 ± 0.37	0.31 ± 0.31	−	−	−
SS8	0.95 ± 0.37	0.43 ± 0.38	0.29 ± 0.38	−	−	−
SS9	0.99 ± 0.35	0.51 ± 0.35	0.32 ± 0.33	−	−	−
SS10	1.57 ± 0.35	1.45 ± 0.38	1.31 ± 0.32	1.14 ± 0.12	0.79 ± 0.27	0.47 ± 0.22
SS11	0.97 ± 0.34	0.61 ± 0.37	0.42 ± 0.38	0.22 ± 0.18	−	−
SS12	0.89 ± 0.23	0.63 ± 0.36	0.34 ± 0.36	−	−	−
SS13	0.87 ± 0.31	0.39 ± 0.36	0.21 ± 0.32	−	−	−
SS14	0.79 ± 0.29	0.42 ± 0.39	0.27 ± 0.34	−	−	−
SS15	0.76 ± 0.26	0.31 ± 0.35	−	−	−	−
SS16	0.85 ± 0.27	0.65 ± 0.32	0.19 ± 0.11	−	−	−
SS17	0.88 ± 0.29	0.74 ± 0.31	0.34 ± 0.13	−	−	−
SS18	1.49 ± 0.28	1.41 ± 0.33	1.32 ± 0.21	1.15 ± 0.19	0.91 ± 0.21	0.56 ± 0.21
SS19	1.32 ± 0.22	1.09 ± 0.35	0.57 ± 0.22	0.23 ± 0.17	−	−
SS20	1.25 ± 0.25	0.97 ± 0.38	0.45 ± 0.23	0.26 ± 0.15	−	−

*SS, Sewage sludge samples; No growth (−).

**Table 2:** Bacterial isolates from sewage sludge can grow on different concentrations of phenol.

Isolates	Concentrations of Phenol (mg/L)					
	Control	500	750	1000	1500	2000
S-1	+++	++	+	−	−	−
S-2	+++	++	++	+	−	−
S-3	+++	+++	+++	+++	++	+
S-4	+++	++	+	−	−	−
S-5	+++	+	+	−	−	−
S-6	+++	++	++	+	−	−
S-7	+++	+	+	−	−	−
S-8	+++	+	+	−	−	−
S-9	+++	+	+	−	−	−
S-10	+++	+++	+++	+++	++	+
S-11	+++	++	++	+	−	−
S-12	+++	+	+	−	−	−
S-13	+++	+	+	−	−	−
S-14	+++	++	+	−	−	−
S-15	+++	+	−	−	−	−
S-16	+++	++	+	−	−	−
S-17	+++	+	+	−	−	−
S-18	+++	+++	+++	+++	++	+
S-19	+++	++	+	−	−	−
S-20	+++	+	+	−	−	−

Very good (+++) Good growth (++) Moderate (+) No growth (−).

**Table 3:** Preliminary identification of bacterial isolate by conventional microbiological methods.

Characteristics	Bacterial Isolates		
	S3	S10	S18
Colonial Characteristics	Circular, White	White Milk	White
Morphological characters			
Gram’s reaction	−	−	−
Shape cell	Rod	Rod	Rod
Spore staining	−	−	−
Motility test (36 °C)	+	−	−
Biochemical characters			
Indole (convert Trp to indole)	−	−	−
Methyl red (Glu fermentation)	−	−	−
Voges–Proskauer (Glu fermentation)	−	+	+
Citrate utilization	−	+	+
Catalase	−	+	+
Oxidase	−	−	−
Nitrate reduction	−	+	+
Urease test	−	+	+
Lysine	+/−	+	+
Arginine	+	−	−
Glucose fermentation	+	+	+
Lactose fermentation	+	+	+
Maltose fermentation	+	+	+
Sucrose fermentation	+	+	+

Trp, tryptophane; Glu, glucose.

**Table 4:** Effect of *Pseudomonas aeruginosa*, *Klebsiella variicola*, and *Klebsiella pneumonia* on germination and seedling growth (Mean ± SD) of different beans at phenol concentration regimes.

		Soybean		Faba Bean		Bean	
Treatment	Phenol Concentration (mg/L)	Germ. of Seeds (%)	Seedling Length (cm)	* Germ. of Seeds (%)	Seedling Length (cm)	Germ. of Seeds (%)	Seedling Length (cm)
Water	0.0	100	2.83 ± 0.65	100	1.88 ± 0.45	100	1.95 ± 0.23
Control	250	0	0	0	0	0	0
	500	0	0	0	0	0	0
	750	0	0	0	0	0	0
	1000	0	0	0	0	0	0
*P. aeruginosa*							
	250	100	2.83 ± 0.45	100	1.78 ± 0.37	100	1.94 ± 0.68
	500	100	2.68 ± 0.34	100	1.58 ± 0.65	100	1.74 ± 0.48
	750	100	2.52 ± 0.35	100	1.48 ± 0.35	100	1.64 ± 0.28
	1000	60	2.41 ± 0.24	60	1.41 ± 0.41	60	1.45 ± 0.37
*K. variicola*							
	250	100	2.98 ± 0.32	100	1.98 ± 0.23	100	1.84 ± 0.26
	500	100	2.77 ± 0.24	100	1.92 ± 0.31	100	1.74 ± 0.29
	750	100	2.68 ± 0.61	100	1.91 ± 0.36	100	1.64 ± 0.23
	1000	100	2.58 ± 0.22	100	1.82 ± 0.52	100	1.54 ± 0.26
*K. pneumoniae*							
	250	100	1.88 ± 0.25	100	1.28 ± 0.42	100	1.34 ± 0.52
	500	100	1.85 ± 0.61	100	1.22 ± 0.47	100	1.32 ± 0.45
	750	60	1.81 ± 0.35	60	1.08 ± 0.25	60	1.31 ± 0.43
	1000	40	1.23 ± 0.25	50	1.01 ± 0.62	50	1.21 ± 0.25

* Germ: Germination.

## Data Availability

Not applicable.

## Supplementary Materials

The following supporting information can be downloaded at: https://www.mdpi.com/article/10.3390/molecules28031203/s1, Figure S1: HPLC analysis for phenol-degrading bacterial strains of *Pseudomonas aeruginosa* (S3), *Klebsiella pneumoniae* (S10), and *Klebsiella variicola* (S18).

## Sample Availability

Samples of the compounds and identified strains are available from the authors.
