# Supplementary material for: Characterization and Biodegradation of Phenol by Pseudomonas aeruginosa and Klebsiella variicola Strains Isolated from Sewage Sludge and Their Effect on Soybean Seeds Germination
Source: Molecules. 2023 Jan 26;28(3):1203. doi: 10.3390/molecules28031203 (PMC9921572; doi:10.3390/molecules28031203)
Supplement: Supplementary file 1 [file molecules-28-01203-s001.zip › molecules-2085450-supplementary.pdf]

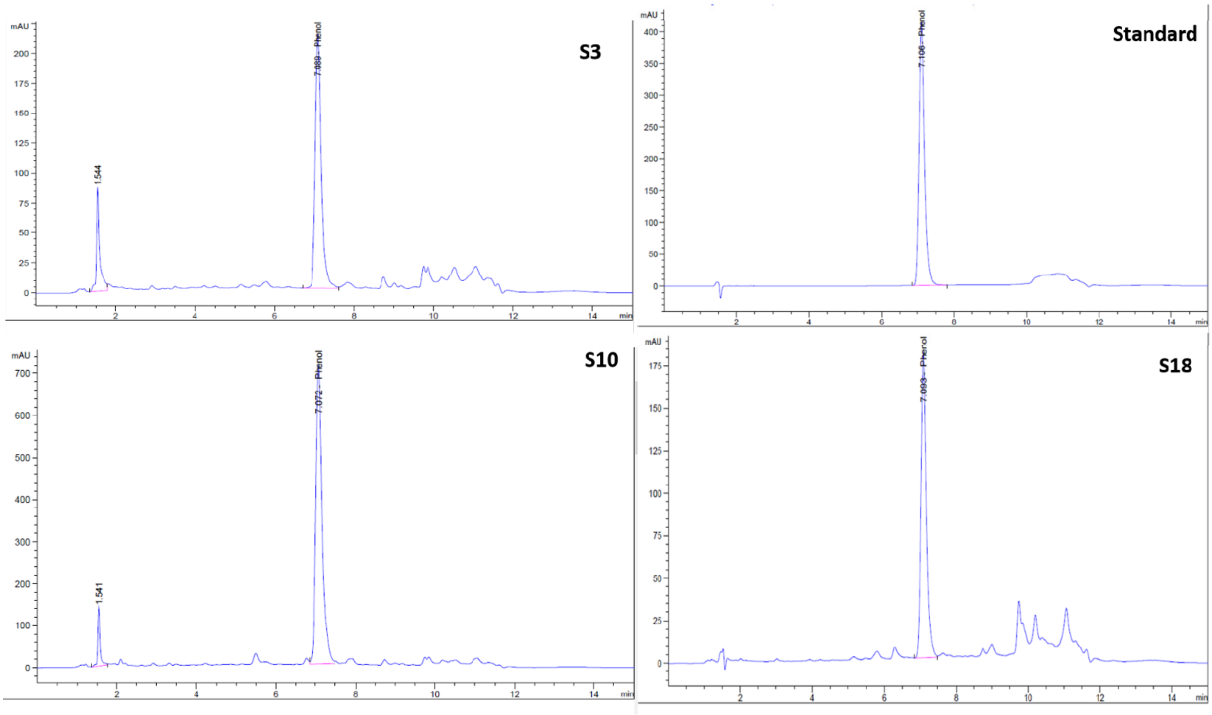

Figure S1: HPLC analysis for phenol-degrading bacterial strains of *Pseudomonas aeruginosa* (S3), *Klebsiella pneumoniae* (S10), and *Klebsiella variicola* (S18).
